# Joint effects of diabetic-related genomic loci on the therapeutic efficacy of oral anti-diabetic drugs in Chinese type 2 diabetes patients

**DOI:** 10.1038/srep23266

**Published:** 2016-03-17

**Authors:** Miao Chen, Rong Zhang, Feng Jiang, Jie Wang, Danfeng Peng, Jing Yan, Shiyun Wang, Tao Wang, Yuqian Bao, Cheng Hu, Weiping Jia

**Affiliations:** 1 Shanghai Diabetes Institute, Department of Endocrinology and Metabolism, Shanghai Clinical Center for Diabetes, Shanghai Key Clinical Center for Metabolic Disease, Shanghai Key Laboratory of Diabetes Mellitus, Shanghai Jiao Tong University Affiliated Sixth People’s Hospital, 600 Yishan Road, Shanghai, 200233, China; 2 Shanghai Jiao Tong University Affiliated Sixth People’s Hospital South Campus, 6600 Nanfeng Road, Shanghai, 201499, China

## Abstract

Previous pharmacogenomic studies of oral anti-diabetic drugs have primarily focused on the effect of a single site. This study aimed to examine the joint effects of multiple loci on repaglinide or rosiglitazone efficacy in newly diagnosed type 2 diabetes mellitus (T2DM) patients. A total of 209 newly diagnosed T2DM patients were randomly assigned to treatment with repaglinide or rosiglitazone for 48 weeks. The reductions in fasting glucose (ΔFPG), 2h glucose (Δ2hPG) and glycated hemoglobin (ΔHbA1c) levels were significantly associated with genetic score that was constructed using the sum of the effect alleles both in the repaglinide (*P* = 0.0011, 0.0002 and 0.0067, respectively) and rosiglitazone cohorts (*P* = 0.0002, 0.0014 and 0.0164, respectively) after adjusting for age, gender, body mass index and dosage. Survival analyses showed a trend towards a greater attainment rate of target HbA1c level in individuals with a high genetic score in the repaglinide cohort and rosiglitazone cohort (*P*_*log-rank*_ = 0.0815 and 0.0867, respectively) when the attainment of treatment targets were defined as more than 20% decrease of FPG, 2hPG, and HbA1c levels after treatment. In conclusion, we identified the joint effects of several T2DM-related loci on the efficacy of oral anti-diabetic drugs; moreover, we built a model to predict the drug efficacy.

Type 2 diabetes mellitus (T2DM) is a worldwide epidemic and chronic disease with high mortality and morbidity. Long-term poorly controlled blood glucose levels contribute to the development of micro- and macro-vascular complications. Consequently, glycemic control is the most imperative strategy in the treatment of T2DM^1^. However, although various oral anti-diabetic drugs are available today, there are often poor therapeutic outcomes in clinical treatment, which may be a result of the overlook of inter-individual variability in responses to drugs.

In addition to factors such as age, gender, severity of disease, hepatic and renal functions, and drug interactions, genetic variations also play an important role in the individual variability of drug response^2^. Pharmacogenomic studies have demonstrated that the variations in genes involved in drug absorption, transport, metabolism and action could have an effect on the pharmacokinetics or pharmacodynamics of drugs^3^,^4^. In addition, with the progress of genome-wide association studies (GWASs), over 90 susceptibility loci for T2DM have been identified. These loci might also have impact on the efficacy of insulin secretagogue and/or sensitizer since they affected insulin secretion and/or sensitivity. A common variant, *PSMD6* rs831571^5^ as an example, might be associated with the efficacy of both insulin secretagogue and sensitizer.

We chose two categories of anti-diabetic drugs: insulin secretagogue (repaglinide) and insulin sensitizer (rosiglitazone). Repaglinide is a member of glinides, which are a type of fast-acting insulin secretagogue. It initiates insulin secretion by closing the ATP-dependent potassium channels and mimics the early rise in insulin concentration, thus lowing plasma glucose levels especially the postprandial glucose. Rosiglitazone is insulin-sensitizing agent which acts as agonists of the nuclear factor peroxisome proliferator–activated receptor γ (PPARγ), thus leading to the improvements of the insulin sensitivity. Many variants of T2DM-related loci had been identified associated with the response of these two drugs in the previous studies, including *PSMD6*, *KCNJ11*, *ABCA1*, *SLC30A8*, *UCP2*, *KCNQ1*, *PAX4* and *NOS1AP*^5^,^6^,^7^,^8^,^9^,^10^,^11^,^12^. However, most of the studies focused only on the effect of a single site on drug efficacy. T2DM is a polygenic complex disease. As a result, the effect of a single site on drug efficacy might be limited, thus making the consideration of a single variant on drug efficacy insufficient in personalized medicine. We therefore conducted this study to examine the joint effects of T2DM-related loci on the efficacy of repaglinide and rosiglitazone in newly diagnosed patients with T2DM.

## Results

### Repaglinide cohort

A total of 91 patients who were treated with repaglinide completed the 48-week study. Among the 13 patients who withdrew from the study, 4 had a glycated haemoglobin (HbA1c) level ≥8% at two consecutive time points, and 9 patients were lost to follow-up. Of the 46 single-nucleotide polymorphisms (SNPs) that were genotyped, 2 SNPs (rs7957197 and rs8042680) were excluded because of departure from Hardy–Weinberg equilibrium (*P* < 0.01).

The associations between the single variant and the reduction in plasma glucose and HbA1c levels after 48 weeks of treatment are shown in supplementary Tables S1–3. Twenty-two SNPs that had been identified affect repaglinide efficacy and met any of the following conditions were selected for inclusion into the combined analysis: a beta value in the linear regression of differences of fasting plasma glucose after 48 weeks treatment (ΔFPG) ≤ −0.5 mmol/L, differences of 2h plasma glucose (Δ2hPG) ≤−1 mmol/L or differences of glycated haemoglobin (ΔHbA1c) ≤ −0.5%, or the P values < 0.2 (shown in Table 1).

**Figure 1:**
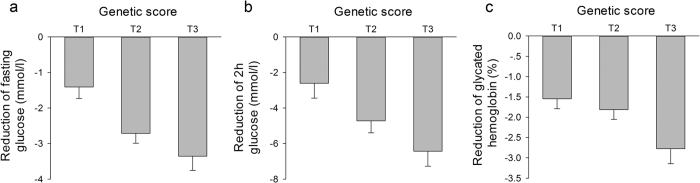
Reduction of fasting glucose, 2h glucose and glycated hemoglobin levels among tertiles of the genetic score in the repaglinide cohort. Linear regressions under an additive model with adjustment for age, gender, BMI and dosage were adopted. The genetic scores of T1, T2 and T3 groups were ≦12, 13–15, ≧16, respectively. (**a**) *P* = 0.0011, β ± SE = −0.85 ± 0.25; **(b)** *P* = 0.0002, β ± SE = −2.09 ± 0.52; **(c)** *P* = 0.0067, β ± SE = −0.60 ± 0.22.

The changes of plasma glucose and HbA1c levels were compared among tertiles of the genetic score (shown in Fig. 1). We found that the reductions in FPG, 2hPG and HbA1c levels after 48 weeks of treatment with repaglinide were significantly associated with the genetic score of SNPs after adjusting for age, gender and body mass index (BMI) (*P* = <0.0001, 0.0001 and 0.0102, respectively; β ± SE = −1.05 ± 0.23, −2.17 ± 0.53, and −0.68 ± 0.26, respectively). Besides adjusting for the common confounding factors as age, gender and BMI, we also adjusted for the baseline FPG, 2hPG or HbA1c respectively to compare the reductions in FPG, 2hPG and HbA1c levels among tertiles of the genetic score. We found the trends for the reductions in FPG, 2hPG and HbA1c still exist (*P* = 0.0033, 0.0158 and 0.0574, respectively; β ± SE = −0.58 ± 0.19, −1.03 ± 0.42, and −0.23 ± 0.12, respectively). In addition, we also adjusted for the dosage, as well as age, gender and BMI at baseline, the reductions in FPG, 2hPG and HbA1c levels were still significantly associated with the genetic score of SNPs (*P* = 0.0011, 0.0002 and 0.0067, respectively; β ± SE = −0.85 ± 0.25, −2.09 ± 0.52, and −0.60 ± 0.22, respectively). Individuals with a high genetic score showed a greater reduction in FPG, 2hPG and HbA1c levels.

**Figure 2:**
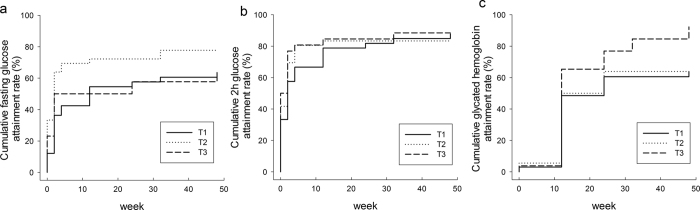
Associations between the attainment rate of the target levels of fasting glucose, 2h glucose and glycated haemoglobinamong tertiles ofthe genetic score of SNPsin the repaglinide cohort. (**a**) *P*_log-rank_ = 0.0545, *P*_Cox-regression_ = 0.7348; (**b)** *P*_log-rank_ = 0.591, *P*_Cox-regression_ = 0.3244; and (**c)** *P*_log-rank_ = 0.0815, *P*_Cox-regression_ = 0.0829. The *P*_Cox-regression_ values were adjusted for age, gender, and BMI at baseline.

Then we adopted survival analyses to explore the association between the attainment rate of the target levels and the genetic score of the SNPs. The withdrawals owning to inadequately controlled blood glucose or glycated hemoglobin were defined as non-responders in the analyses. When the attainments of treatment targets were defined as glycemic thresholds, there were no significant differences in the attainment rate of FPG, 2hPG and HbA1c levels among the tertiles of the genetic score (*P*_*log-rank*_ = 0.0567, 0.6348 and 0.5455, respectively). When more than 20% decrease of FPG, 2hPG, and HbA1c levels after treatment compared to the baseline were defined as attainment, the survival analyses showed that there was a trend towards a greater attainment rate of HbA1c in individuals with a high genetic score (*P*_*log-rank*_ = 0.0815) (Fig. 2c). The attainment rate of HbA1c in patients with the highest tertile of the genetic score was significantly higher than in patients with the lowest tertile of the genetic score (*P* = 0.023). A Cox regression adjusting for age, gender, and BMI at baseline also revealed that patients with a high genetic score were more likely to attain the standard HbA1c level (*P* = 0.0829).

### Rosiglitazone cohort

Of a total of 105 patients who were treated with rosiglitazone, 93 patients completed the 48-weeks follow-up. Twelve patients withdrew from the study, including one patient who had elevated hepatic enzymes, 5 patients who had inadequate control of blood glucose levels, and 6 patients who were lost to follow-up. One patient was excluded because of failure to be genotyped. Therefore, 92 patients were ultimately included in the statistical analysis. All of the SNPs were in agreement with Hardy–Weinberg equilibrium except for rs864745 and rs7957197 (*P* < 0.01).

The associations between the SNPs and Δvalues of plasma glucose and HbA1c levels after 48 weeks of rosiglitazone treatment are shown in supplementary Tables S4–6. Of the 46 genotyped SNPs, 23 SNPs were selected into the combined analysis according to the criteria mentioned above (shown in Table 1).

**Figure 3:**
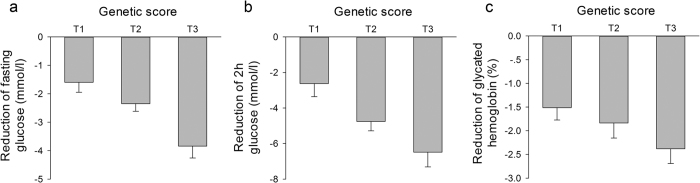
Reduction of fasting glucose, 2h glucose and glycated haemoglobin levels among tertiles of the genetic score in the rosiglitazone cohort. Linear regressions under an additive model with adjustment for age, gender, BMI and dosage were adopted. The genetic scores of T1, T2 and T3 groups were ≦21, 22–24, ≧25, respectively. **(a)** *P* = 0.0002, β ± SE = −1.13 ± 0.28; **(b)** *P* = 0.0014, β ± SE = −1.94 ± 0.58; **(c)** *P* = 0.0164, β ± SE = −0.56 ± 0.23.

The reductions in the FPG, 2hPG and HbA1c levels were compared among tertiles of the genetic score (shown in Fig. 3). The results showed that the reductions in the FPG, 2hPG and HbA1c levels after 48 weeks of treatment were significantly associated with the genetic score of the SNPs after adjusting for age, gender and BMI (*P* = <0.0001, 0.0018 and 0.0479, respectively; β ± SE = −1.09 ± 0.26, −1.78 ± 0.55 and −0.46 ± 0.23, respectively). Besides adjusting for the common confounding factors as age, gender and BMI, we also adjusted for the baseline FPG, 2hPG or HbA1c respectively to compare the reductions in FPG, 2hPG and HbA1c levels among tertiles of the genetic score. We found the trends for the reductions in FPG, 2hPG and HbA1c still exist (*P* = 0.2807, 0.0748 and 0.1842, respectively; β ± SE = −0.20 ± 0.19, −0.68 ± 0.38, and −0.17 ± 0.12, respectively). In addition, after adjusting for the dosage, as well as age, gender and BMI at baseline, the reductions in FPG, 2hPG and HbA1c levels were still significantly associated with the genetic score of SNPs (*P* = 0.0002, 0.0014 and 0.0164, respectively; β ± SE = −1.13 ± 0.28, −1.94 ± 0.58, and −0.56 ± 0.23, respectively). Individuals with a high genetic score showed a greater reduction in FPG, 2hPG and HbA1c levels.

**Figure 4:**
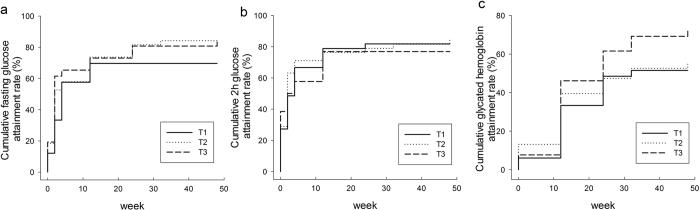
Associations between the attainment rate of the target levels of fasting glucose, 2h glucose and glycated haemoglobinamong tertiles ofthe genetic score of SNPs in the rosiglitazone cohort. (**a**) *P*_log-rank_ = 0.1477, *P*_Cox-regression_ = 0.0654; (**b)** *P*_log-rank_ = 0.7902, *P*_Cox-regression_ = 0.8224; and (**c)** *P*_log-rank_ = 0.0867, *P*_Cox-regression_ = 0.0811. The *P*_Cox-regression_ values were adjusted for age, gender, and BMI at baseline.

When the attainments of treatment targets were defined as glycemic thresholds, there were no significant differences in the attainment rate of FPG, 2hPG and HbA1c levels among the tertiles of the genetic score (*P*_*log-rank*_ = 0.4917, 0.9376 and 0.9179, respectively). When more than 20% decrease of FPG, 2hPG, and HbA1c levels after treatment compared to the baseline were defined as attainment, the survival analyses showed there was a trend towards a greater attainment rate of HbA1c in individuals with a high genetic score (*P*_*log-rank*_ = 0.0867) (Fig. 4c). The attainment rate of HbA1c in patients with the highest tertile of the genetic score was significantly higher than in patients with lowest tertile of the genetic score (*P* = 0.024). A Cox regression also showed that patients with high genetic score were more likely to attain the standard HbA1c levels (*P* = 0.0811).

## Discussion

In the present study, we identified the joint effects of several T2DM-related loci on the therapeutic efficacy of oral anti-diabetic drugs. We compared the glycemic control among the tertiles of genetic score, and found the reduction in glycemia and cumulative attainment rate of target levels after repaglinide or rosiglitazone treatment were associated with the genetic scores of the SNPs. When the genetic score was treated as a continuous variable, the associations between the reduction of glycemia and genetic score still existed. Patients with a high genetic score were more likely to attain better glycemic control.

The inter-individual variability in the outcomes of the hypoglycemic agents was largely attributed to the different genetic background of the patients. Hence, a great deal of attention should be paid to genetic factors when optimizing of an appropriate therapy to improve drug efficacy, to decrease the number of therapy failures and to reduce the incidence of T2DM complications. In recent years, pharmacogenomic studies have offered increasingly important and useful information to help us understand the effects of genetic factors. More and more variants have been identified along with their influence on clinical responses to anti-diabetic drugs. For instance, the *KCNQ1* gene, which confers the strongest risks with T2DM in East Asians^13^,^14^, has been found associated with repaglinide and rosiglitazone efficacy^15^,^16^. Nevertheless, the genetic effect of single locus is still limited due to the heterogeneity and complexity in genetic susceptibility to T2DM. The evaluation of joint effects of multiple loci is necessary. The combined analysis and genetic risk score have been widely applied to explore the association between genetic background and incidence of diseases^17^,^18^,^19^,^20^. However, few pharmacogenetic studies adopted the method of genetic score to evaluate the joint effects of multiple loci on drug efficacy^21^. In this study, we first integrated the effects of multiple genes and evaluated the correlation between the genetic score and drug efficacy. The model we developed in this study greatly improves the accuracy of efficacy prediction over the single site effect and provides useful information in drug selection, dose titration and treatment duration. The development of this model offers the possibility of personalizing the treatment of T2DM.

We adopted two criteria to define the attainment of treatment targets in this study. Despite it is more convenient to adopt glycemic thresholds as criterion, using more than 20% decrease of plasma glucose or HbA1c levels after treatment take into account the effects of baseline plasma glucose on drug efficacy evaluation. We did not find significant difference in the attainment rate of FPG, 2hPG and HbA1c levels among the tertiles of the genetic score when using glycemic thresholds to define attainment. This might be attributed to the high attainment rate of FPG, 2hPG and HbA1c of all the tertiles of the genetic score under this criterion.

Although our study is meaningful for personalized medicine of T2DM, there are several limitations that should be noted. First, in addition to genetic factors, other environmental factors such as education, compliance to medication, access to health care and physical exercise, as well as the biological factors of patients, such as age, gender, and kidney and liver function, also attribute to the individual variability of drug efficacy^22^. The model we developed did not take into account the effect of these non-genetic factors. In future studies, this model will be improved by taking these additional factors into account. Second, we have not conducted a prospective study to validate the predictive value of the drugs’ efficacy and feasibility in guiding drug prescriptions. This prospective study is required in the future. Third, because the mechanism underlying the association between the loci we investigated and the incidence of T2DM remains unknown, we are unsure about the underlying molecular mechanism of their pharmacogenomic effects. Therefore, more functional studies will be performed to investigate this in the future. Fourth, the sample size of this study was relatively small; consequently, we may not have had enough statistical power to detect effects of genetic variants on some of the parameters. In addition, our study did not include all of the susceptibility loci for T2DM known so far. With our knowledge about the pathogenesis of T2DM that are currently known, more and more loci will be supplied into this model.

In conclusion, we identified the joint effects of several T2DM-related locion the therapeutic efficacy of oral anti-diabetic drugs; moreover, we built a model to predict drug efficacy. Although this study offers the possibility of applying personalized medicine in T2DM, more prospective and functional studies are required in the future to confirm the value of this model.

## Methods

### Patients and study design

A total of 209 newly diagnosed T2DM patients who were diagnosed on the basis of World Health Organization criteria were recruited from outpatient clinics in Shanghai, China. None of the patients had received any anti-hyperglycemic therapies prior to the study. Detailed information on this study population has been described previously^23^,^24^. After a 2-week run-in period, all of the subjects were randomly assigned to treatment with either repaglinide (NovoNorm; Novo Nordisk, Copenhagen, Denmark, n = 104) or rosiglitazone (Avandia; GlaxoSmithKline, Munich, Germany, n = 105) for 48 weeks. The patients were visited at weeks 0, 2, 4, 12, 24, 32, and 48 and underwent the designed clinical assessments. The medication doses were titrated according to the blood glucose levels during the visits. Repaglinide or rosiglitazone was administered initially in a mealtime dosage of 0.5 mg or 4mg, respectively. The dosage was increased stepwise to 1, 1.5, and 2 mg for repaglinide and to 8mg for rosiglitazone until the patients achieved the glycemic targets of fasting plasma glucose <7 mmol/L (126 mg/dL) and/or 2 h plasma glucose <11 mmol/L (200 mg/dL). Patients with FPG > 13 mmol/L (234 mg/dL), 2hPG > 18 mmol/L (324 mg/dL) or HbA1c ≥8% at 2 consecutive time points (a maximal interval of 6 d) were excluded from the study.

This study was approved by the institutional review board of the Shanghai Jiao Tong University Affiliated Sixth People’s Hospital, Shanghai, China. Written informed consent was obtained from each patient. All of the experiments were conducted according to the guidelines and the regulations of the Ethical Committee of Shanghai Jiao Tong University Affiliated Sixth People’s Hospital.

### Anthropometric and clinical laboratory measurements

Anthropometric parameters including height (m), weight (kg), and blood pressure (mmHg) were measured in all of the patients at baseline and at 48 weeks after repaglinide or rosiglitazone treatment. BMI (kg/m2) was calculated as weight/height^2^.

Overnight fasting and 2 hour blood samples after a 75 g oral glucose tolerance test (OGTT) were collected at each visit. Plasma glucose concentrations were measured by means of the glucose oxidase-peroxidase method, using commercial kits (Shanghai Biological Products Institution, Shanghai, China). HbA1c values were measured by means of high-performance liquid chromatography, using a Bio-Rad Variant II haemoglobin testing system (Bio-Rad Laboratories, Hercules, CA).

### SNP selection and genotyping

We selected 46 SNPs that have been previously reported to be associated with T2DM at the genome-wide associated level^15^,^25^,^26^,^27^,^28^. We also selected 2 variants of genes that are mainly responsible for repaglinide or rosiglitazone metabolism (CYP2C8*3 (rs7098376), CYP3A4*18 (rs12721629))^29^,^30^. However, because the genotypes of these two variants were monomorphic according to our results, they were excluded in the statistical analysis. Genomic DNA was extracted from peripheral blood leucocytes in the whole blood samples. All of the selected SNPs were genotyped by means of matrix-assisted laser desorption/ionization time-of-flight mass spectroscopy using a MassARRAY platform (MassARRAY Compact Analyser; Sequenom, San Diego, CA) or DNA sequencing using 3130xl Genetic Analyser (Applied Biosystem, Foster City, CA, USA). The genotyping results were confirmed by DNA sequencing using a 3130xl Genetic Analyser (Applied Biosystem, Foster City, CA, USA).

### Statistical analysis

Hardy–Weinberg equilibrium tests were performed for each SNP in the repaglinide or rosiglitazone cohort using x^2^ tests. The data are shown as mean values ± SEM. Δvalues were calculated as the final value at 48 weeks minus the initial value. A linear regression under an additive model with adjustment for age, gender and BMI at baseline was used to test the association between the SNPs and differences of quantitative traits.

The SNPs that were had been found to have an effect on the drug efficacy were selected for the combined analysis when they met the any of following conditions: the beta value of the linear regression mentioned above was ΔFPG ≤ −0.5 mmol/L, Δ2hPG ≤ −1 mmol/L or ΔHbA1c ≤ −0.5%, or P values < 0.2. A genetic score was constructed using the sum of effect alleles of selected SNPs showed in Table 1. Individual SNP was recoded as 0, 1, and 2 according to the number of effect alleles which showed negative beta values in the linear regression. Genetic score = SNP_1_ + SNP_2_ + … + SNP_n._ The differences of quantitative traits were compared among tertiles of the genetic score using a linear regression under an additive model with adjustment for age, gender, and BMI at baseline.

There were two definitions of attainment of treatment targets: 1. FPG < 7.0 mmol/L, 2hPG < 11.1 mmol/L, and HbA1c < 7.0% were defined as attainment of FPG, 2hPG and HbA1c, respectively. 2. The decrease of FPG, 2hPG, and HbA1c levels by more than 20% after treatment compared to the baseline were defined as attainment. The withdrawals owning to inadequately controlled blood glucose or glycated hemoglobin were defined as non-responders in the analysis. Attainment rates among tertiles of the genetic risk score were compared by means of the log-rank test and a Cox regression model analysis with adjustment for confounding factors, namely, age, gender, and BMI at baseline. A two-tailed P value of <0.05 was considered statistically significant. The statistical analyses were performed using PLINK (v1.07) or SAS for Windows (version 8.0; SAS Institute, Cary, NC).

## Additional Information

**How to cite this article**: Chen, M. *et al.* Joint effects of diabetic-related genomic loci on the therapeutic efficacy of oral anti-diabetic drugs in Chinese type 2 diabetes patients. *Sci. Rep.* **6**, 23266; doi: 10.1038/srep23266 (2016).

## Supplementary Material

Supplementary Information

## Figures and Tables

**Table 1:** Selected SNPs for the genetic score calculation.

Repaglinide						
Gene/location	SNP	Chromosome	Effect allele	BETA±SE (ΔFPG)	BETA±SE (Δ2hPG)	BETA±SE (ΔHbA1c)
*PPARG*	rs1801282	3	G	−1.13 ± 0.71	−2.92 ± 1.62	−1.22 ± 0.62
*IGF2BP2*	rs7651090	3	G	–	−1.22 ± 0.85	–
*WFS1*	rs10010131	4	A	–	−2.05 ± 1.79	–
*ZBED3-AS1*	rs4457053	5	G	−0.53 ± 0.77	–	–
*CDKAL1*	rs7756992	6	A	−0.85 ± 0.34	–	−0.55 ± 0.29
*KCNK16*	rs1535500	6	G	–	−1.11 ± 0.69	–
*YKT6*	rs917793	7	T	−0.73 ± 0.38	–	–
*KLF14*	rs972283	7	A	–	−1.3 ± 0.85	–
*JAZF1*	rs864745	7	G	–	−1.25 ± 0.72	–
*PAX4*	rs6467136	7	A	–	–	−0.55 ± 0.27
*PTPRD*	rs17584499	9	T	−0.53 ± 0.48	–	–
*VPS26A*	rs1802295	10	C	−0.5093 ± 0.51	–	−0.70 ± 0.42
*TCF7L2*	rs7903146	10	T	−0.68 ± 0.56	–	−0.94 ± 0.48
*ARAP1*	rs1552224	11	G	−0.66 ± 0.49	−1.03 ± 1.13	–
*KCNJ11*	rs5219	11	T	–	–	−0.60 ± 0.26
*TENM4*	rs10751301	11	C	–	−1.14 ± 0.88	–
*TSPAN8*	rs7961581	12	T		−1.04 ± 0.82	
13q31.1	rs1359790	13	T	−0.57 ± 0.34	−1.20 ± 0.77	–
*ZFAND6*	rs11634397	15	G	−0.79 ± 0.64	−1.80 ± 1.40	–
*AP3S2*	rs2028299	15	A	−0.68 ± 0.32	–	–
*MAF*	rs17797882	16	T	−0.52 ± 0.35	–	–
*SRR*	rs391300	17	A	−0.52 ± 0.36	−1.39 ± 0.86	−0.70 ± 0.29
**Rosiglitazone**						
**Gene**	**SNP**	**Chromosome**	**Effect allele**	**BETA** ± **SE (ΔFPG)**	**BETA** ± **SE (Δ2hPG)**	**BETA** ± **SE (ΔHbA1c)**
*RBMS1*	rs7593730	2	T	−0.52 ± 0.50	–	–
near *GRB14*	rs3923113	2	C	−1.38 ± 0.45	–	–
*UBE2E2*	rs7612463	3	A	−0.59 ± 0.40	−1.14 ± 0.78	–
*PPARG*	rs1801282	3	G	−1.00 ± 0.73	−2.68 ± 1.39	–
*MAEA*	rs6815464	4	C	−0.54 ± 0.35	–	–
*WFS1*	rs10010131	4	A	–	−1.30 ± 1.57	–
*ZBED3-AS1*	rs4457053	5	A	–	−2.40 ± 1.49	–
*ZFAND3*	rs9470794	6	C	−0.54 ± 0.34	–	–
*YKT6*	rs917793	7	A	−0.62 ± 0.34	–	–
*PAX4*	rs6467136	7	A	–	−2.04 ± 0.84	–
*TP53INP1*	rs896854	8	G	–	–	−0.52 ± 0.27
*SLC30A8*	rs13266634	8	T	–	−1.16 ± 0.60	–
*PTPRD*	rs17584499	9	C	–	−2.18 ± 1.23	−0.76 ± 0.52
*CDC123*	rs12779790	10	A	−0.63 ± 0.37	–	–
*TCF7L2*	rs7903146	10	C	–	−1.50 ± 1.64	–
*KCNQ1*	rs231362	11	C	–	−1.08 ± 0.94	–
*ARAP1*	rs1552224	11	G	–	–	−0.52 ± 0.49
*TENM4*	rs10751301	11	G	–	−1.53 ± 0.73	–
13q31.1	rs1359790	13	C	–	−0.71 ± 0.67	–
*HMG20A*	rs7178572	15	A	−0.57 ± 0.28	–	−0.50 ± 0.22
*ZFAND6*	rs11634397	15	G	−0.56 ± 0.56	−1.04 ± 1.10	–
*MAF*	rs17797882	16	C	–	−1.02 ± 0.64	–
*FTO*	rs8050136	16	C	−0.68 ± 0.37	–	–

Note: Linear regression was under an additive model with adjustment for age, gender, BMI at baseline.
