# Supplementary material for: Joint effects of diabetic-related genomic loci on the therapeutic efficacy of oral anti-diabetic drugs in Chinese type 2 diabetes patients
Source: Sci Rep. 2016 Mar 17;6:23266. doi: 10.1038/srep23266 (PMC4794654; doi:10.1038/srep23266)
Supplement: Supplementary Information [file srep23266-s1.pdf]

Joint effects of diabetic-related genomic loci on the therapeutic efficacy of oral antidiabetic drugs in Chinese type 2 diabetes patients

Miao Chen[1*], Rong Zhang[1*], Feng Jiang[1], Jie Wang[1], Danfeng Peng[1], Jing Yan[1], Shiyun Wang[1], Tao Wang[1], Yuqian Bao[1],Cheng Hu[1,2 §], Weiping Jia[1 §]

[1] Shanghai Diabetes Institute, Department of Endocrinology and Metabolism, Shanghai Clinical Center for Diabetes, Shanghai Key Clinical Center for Metabolic Disease, Shanghai Key Laboratory of Diabetes Mellitus, Shanghai Jiao Tong University Affiliated Sixth People's Hospital, 600 Yishan Road, Shanghai, China

[2] Shanghai Jiao Tong University Affiliated Sixth People's Hospital South Campus, Shanghai, China

* These authors contribute equally to this work.

Corresponding author: Weiping Jia

Address: Shanghai Jiao Tong University Affiliated Sixth People's Hospital, 600 Yishan Road, Shanghai, China

Fax: 86-21-64368031 Phone: 86-21-24058260 Email: wpjia@sjtu.edu.cn

Or

Cheng Hu

Address: Shanghai Jiao Tong University Affiliated Sixth People's Hospital, 600 Yishan Road, Shanghai, China

Fax: 86-21-64368031 Phone: 86-21-24058260 Email: alfredhc@sjtu.edu.cn

Supplementary Table 1 Association between SNPs and △FPG after 48 weeks of treatment in the repaglinide cohort

**Gene/location**	**SNP**	**Chromosome**	**Allele**	**BETA**	**SEM**	***P* value**
RBMS1	rs7593730	2	T	-0.2712	0.3956	0.495
near *GRB14*	rs3923113	2	G	0.02082	0.4281	0.9613
UBE2E2	rs7612463	3	A	0.2531	0.3593	0.4832
IGF2BP2	rs7651090	3	G	-0.3772	0.3817	0.326
PPARG	rs1801282	3	G	-1.126	0.7069	0.115
ADAMTS9-AS2	rs4607103	3	T	-0.08306	0.279	0.7667
ST6GAL1	rs16861329	3	T	-0.4168	0.3975	0.2974
MAEA	rs6815464	4	G	0.2819	0.3066	0.3606
WFS1	rs10010131	4	A	-0.1924	0.7653	0.8022
ZBED3-AS1	rs4457053	5	G	-0.5252	0.7617	0.4925
ZFAND3	rs9470794	6	C	0.04615	0.3316	0.8897
CDKAL1	rs7756992	6	A	-0.8483	0.3403	0.01472
KCNK16	rs1535500	6	G	0.2256	0.2868	0.4339
YKT6	rs917793	7	A	0.7348	0.3863	0.06074
KLF14	rs972283	7	A	0.1397	0.3668	0.7043
JAZF1	rs864745	7	G	0.01085	0.3257	0.9735

PAX4	rs6467136	7	A	-0.4611	0.339	0.1776
TP53INP1	rs896854	8	A	0.2924	0.2878	0.3127
SLC30A8	rs13266634	8	T	0.07305	0.2933	0.804
GLIS3	rs7041847	9	G	-0.119	0.3005	0.6932
PTPRD	rs17584499	9	T	-0.5284	0.4763	0.2706
CHCHD9	rs13292136	9	T	-0.02806	0.4965	0.9551
10p13	rs10906115	10	G	0.01443	0.3223	0.9644
VPS26A	rs1802295	10	T	0.5093	0.506	0.3172
CDC123	rs12779790	10	G	0.2588	0.3779	0.4954
TCF7L2	rs7903146	10	T	-0.6795	0.559	0.2277
KCNQ1	rs231362	11	T	0.1038	0.4696	0.8256
ARAP1	rs1552224	11	G	-0.6604	0.4859	0.1779
KCNQ1	rs2237892	11	T	-0.4609	0.3576	0.2011
KCNJ11	rs5219	11	T	-0.1313	0.3225	0.685
TENM4	rs10751301	11	C	-0.4459	0.3602	0.2194
TSPAN8	rs7961581	12	C	0.1645	0.3491	0.6388
OASL	rs7957197	12	T	NA	NA	NA
RPSAP52	rs1531343	12	C	0.1877	0.4925	0.7041

13q31.1	rs1359790	13	T	-0.5657	0.3387	0.09868
HMG20A	rs7178572	15	G	-0.06337	0.3265	0.8466
15q22.2	rs1436955	15	A	-0.03408	0.3365	0.9196
ZFAND6	rs11634397	15	G	-0.7948	0.6395	0.2175
PRC1	rs8042680	15	A	NA	NA	NA
AP3S2	rs2028299	15	C	0.6832	0.3234	0.0377
MAF	rs17797882	16	T	-0.5225	0.3537	0.1434
FTO	rs8050136	16	A	-0.1282	0.4339	0.7684
SRR	rs391300	17	A	-0.5246	0.355	0.1434
PEPD	rs3786897	19	A	-0.04518	0.3198	0.888
HNF4A	rs6017317	20	G	-0.2015	0.2954	0.4971
HNF4A	rs4812829	20	A	0.07394	0.3057	0.8095

Note:

Δ value=T*48 weeks* value - baseline (T0) value. Linear regression was under an additive model with adjustment for age, gender, BMI at baseline.

Supplementary Table 2 Association between SNPs and △2hPG after 48 weeks of treatment in the repaglinide cohort

**Gene/location**	**SNP**	**Chromosome**	**Allele**	**BETA**	**SEM**	***P* value**
RBMS1	rs7593730	2	T	0.005638	0.9004	0.995
near *GRB14*	rs3923113	2	G	-0.04871	0.9631	0.9598
UBE2E2	rs7612463	3	A	-0.3855	0.8487	0.6511
IGF2BP2	rs7651090	3	G	-1.224	0.8529	0.1557
PPARG	rs1801282	3	G	-2.918	1.622	0.07623
ADAMTS9-AS2	rs4607103	3	T	-0.2117	0.6357	0.74
ST6GAL1	rs16861329	3	T	-0.9026	0.898	0.3182
MAEA	rs6815464	4	G	0.2471	0.7123	0.7296
WFS1	rs10010131	4	A	-2.045	1.793	0.2577
ZBED3-AS1	rs4457053	5	G	0.5097	1.782	0.7757
ZFAND3	rs9470794	6	C	0.7876	0.7343	0.287
CDKAL1	rs7756992	6	A	-0.1106	0.8471	0.8964
KCNK16	rs1535500	6	G	1.108	0.6903	0.1127
YKT6	rs917793	7	A	0.4023	0.8953	0.6545
KLF14	rs972283	7	A	-1.299	0.8525	0.1321
JAZF1	rs864745	7	G	-1.25	0.7186	0.08632

PAX4	rs6467136	7	A	-0.2083	0.8024	0.7959
TP53INP1	rs896854	8	A	0.4633	0.6748	0.4946
SLC30A8	rs13266634	8	T	0.2134	0.658	0.7467
GLIS3	rs7041847	9	G	-0.2676	0.6799	0.6951
PTPRD	rs17584499	9	T	-0.9675	1.078	0.3726
CHCHD9	rs13292136	9	T	0.3798	1.092	0.7291
10p13	rs10906115	10	G	-0.2241	0.7278	0.759
VPS26A	rs1802295	10	T	0.8278	1.116	0.4608
CDC123	rs12779790	10	G	-0.1284	0.9264	0.8902
TCF7L2	rs7903146	10	T	-0.7437	1.27	0.56
KCNQ1	rs231362	11	T	-0.6571	1.138	0.5654
ARAP1	rs1552224	11	G	-1.025	1.128	0.3666
KCNQ1	rs2237892	11	T	-0.6908	0.8082	0.3955
KCNJ11	rs5219	11	T	-0.01485	0.7489	0.9842
TENM4	rs10751301	11	C	-1.14	0.8781	0.1985
TSPAN8	rs7961581	12	C	1.036	0.8182	0.2097
OASL	rs7957197	12	T	NA	NA	NA
RPSAP52	rs1531343	12	C	0.782	1.123	0.4884

13q31.1	rs1359790	13	T	-1.203	0.7665	0.1208
HMG20A	rs7178572	15	G	-0.2116	0.7299	0.7727
15q22.2	rs1436955	15	A	0.8286	0.7812	0.2924
ZFAND6	rs11634397	15	G	-1.802	1.397	0.2012
PRC1	rs8042680	15	A	NA	NA	NA
AP3S2	rs2028299	15	C	0.4423	0.77	0.5675
MAF	rs17797882	16	T	-0.4491	0.8082	0.5802
FTO	rs8050136	16	A	2.056	0.9797	0.03933
SRR	rs391300	17	A	-1.393	0.8591	0.1093
PEPD	rs3786897	19	A	0.4319	0.6984	0.5382
HNF4A	rs6017317	20	G	0.03059	0.6775	0.9641
HNF4A	rs4812829	20	A	0.5706	0.6902	0.4111

Note:

Δ value=T*48 weeks* value - baseline (T0) value. Linear regression was under an additive model with adjustment for age, gender, BMI at baseline.

Supplementary Table 3 Association between SNPs and △HbA1c levels after 48 weeks of treatment in the repaglinide cohort

**Gene/location**	**SNP**	**Chromosome**	**Allele**	**BETA**	**SEM**	***P* value**
RBMS1	rs7593730	2	T	-0.4606	0.3283	0.1645
near *GRB14*	rs3923113	2	G	-0.2079	0.3553	0.5601
UBE2E2	rs7612463	3	A	-0.4611	0.297	0.1245
IGF2BP2	rs7651090	3	G	-0.09003	0.3221	0.7806
PPARG	rs1801282	3	G	-1.224	0.6204	0.05195
ADAMTS9-AS2	rs4607103	3	T	0.2679	0.2321	0.2518
ST6GAL1	rs16861329	3	T	-0.2947	0.3318	0.3771
MAEA	rs6815464	4	G	0.216	0.2604	0.4093
WFS1	rs10010131	4	A	0.04686	0.6375	0.9416
ZBED3-AS1	rs4457053	5	G	-0.4687	0.6363	0.4636
ZFAND3	rs9470794	6	C	0.01184	0.2719	0.9654
CDKAL1	rs7756992	6	A	-0.5484	0.2877	0.06021
KCNK16	rs1535500	6	G	0.355	0.2367	0.1377
YKT6	rs917793	7	A	-0.4273	0.3287	0.1973
KLF14	rs972283	7	A	0.09367	0.3084	0.7621
JAZF1	rs864745	7	G	-0.03805	0.272	0.8891

PAX4	rs6467136	7	A	-0.5512	0.2686	0.04341
TP53INP1	rs896854	8	A	0.2055	0.2416	0.3975
SLC30A8	rs13266634	8	T	0.2692	0.2433	0.2717
GLIS3	rs7041847	9	G	0.08832	0.2571	0.7321
PTPRD	rs17584499	9	T	0.1267	0.4057	0.7557
CHCHD9	rs13292136	9	T	0.08342	0.4145	0.841
10p13	rs10906115	10	G	0.03271	0.2705	0.9041
VPS26A	rs1802295	10	T	0.7201	0.4179	0.08875
CDC123	rs12779790	10	G	0.1384	0.3175	0.664
TCF7L2	rs7903146	10	T	-0.939	0.4848	0.05628
KCNQ1	rs231362	11	T	-0.01366	0.3914	0.9722
ARAP1	rs1552224	11	G	-0.412	0.4091	0.317
KCNQ1	rs2237892	11	T	-0.04532	0.3032	0.8815
KCNJ11	rs5219	11	T	-0.5899	0.2634	0.02789
TENM4	rs10751301	11	C	-0.1432	0.3013	0.6359
TSPAN8	rs7961581	12	C	-0.03519	0.2933	0.9048
OASL	rs7957197	12	T	NA	NA	NA
RPSAP52	rs1531343	12	C	0.2245	0.4161	0.591

13q31.1	rs1359790	13	T	-0.3097	0.2853	0.281
HMG20A	rs7178572	15	G	-0.00367	0.2714	0.9893
15q22.2	rs1436955	15	A	0.1824	0.2814	0.5187
ZFAND6	rs11634397	15	G	-0.3643	0.516	0.4822
PRC1	rs8042680	15	A	NA	NA	NA
AP3S2	rs2028299	15	C	0.3893	0.2841	0.1743
MAF	rs17797882	16	T	-0.2188	0.2994	0.4671
FTO	rs8050136	16	A	-0.6026	0.358	0.0962
SRR	rs391300	17	A	-0.7011	0.2919	0.01863
PEPD	rs3786897	19	A	-0.09958	0.265	0.7081
HNF4A	rs6017317	20	G	0.2098	0.25	0.4037
HNF4A	rs4812829	20	A	0.1937	0.2514	0.4434

Note:

Δ value=T*48 weeks* value - baseline (T0) value. Linear regression was under an additive model with adjustment for age, gender, BMI at baseline.

Supplementary Table 4 Association between SNPs and △FPG after 48 weeks of treatment in the rosiglitazone cohort

**Gene/location**	**SNP**	**Chromosome**	**Allele**	**BETA**	**SEM**	***P* value**
RBMS1	rs7593730	2	T	-0.5168	0.4972	0.3018
near *GRB14*	rs3923113	2	G	-1.383	0.4471	0.002736
UBE2E2	rs7612463	3	A	-0.5928	0.3958	0.1382
IGF2BP2	rs7651090	3	G	0.01887	0.3384	0.9557
PPARG	rs1801282	3	G	-1.002	0.7311	0.1745
ADAMTS9-AS2	rs4607103	3	T	0.1003	0.3359	0.7661
ST6GAL1	rs16861329	3	T	0.04641	0.4191	0.9121
MAEA	rs6815464	4	G	0.5355	0.3526	0.1328
WFS1	rs10010131	4	A	0.2011	0.7656	0.7935
ZBED3-AS1	rs4457053	5	G	-0.2005	0.8542	0.8151
ZFAND3	rs9470794	6	C	-0.5447	0.3425	0.1157
CDKAL1	rs7756992	6	A	0.2356	0.3095	0.4486
KCNK16	rs1535500	6	T	-0.3236	0.2996	0.2834
YKT6	rs917793	7	A	-0.6197	0.3396	0.07182
KLF14	rs972283	7	A	-0.2326	0.329	0.4817
JAZF1	rs864745	7	G	-0.337	0.2768	0.2271

PAX4	rs6467136	7	A	-0.06715	0.4474	0.8811
TP53INP1	rs896854	8	A	0.04817	0.35	0.8909
SLC30A8	rs13266634	8	T	0.08165	0.3068	0.7909
GLIS3	rs7041847	9	A	0.0404	0.3188	0.8995
PTPRD	rs17584499	9	T	1.09	0.6394	0.09209
CHCHD9	rs13292136	9	T	0.2517	0.57	0.66
10p13	rs10906115	10	G	0.1954	0.324	0.5482
VPS26A	rs1802295	10	T	-0.05012	0.4053	0.9019
CDC123	rs12779790	10	G	0.6275	0.3712	0.09485
TCF7L2	rs7903146	10	T	-0.413	0.8503	0.6285
KCNQ1	rs231362	11	T	-0.2981	0.4842	0.5399
ARAP1	rs1552224	11	G	0.0746	0.6204	0.9046
KCNQ1	rs2237892	11	T	-0.058	0.3721	0.8765
KCNJ11	rs5219	11	T	0.4872	0.3027	0.1116
TENM4	rs10751301	11	C	0.4126	0.3617	0.2575
TSPAN8	rs7961581	12	C	0.05681	0.3437	0.8691
OASL	rs7957197	12	T	NA	NA	NA
RPSAP52	rs1531343	12	C	-0.07591	0.5231	0.885

13q31.1	rs1359790	13	T	0.7057	0.3285	0.03476
HMG20A	rs7178572	15	G	0.5732	0.2788	0.04308
15q22.2	rs1436955	15	A	0.1366	0.3451	0.6932
ZFAND6	rs11634397	15	G	-0.5586	0.5561	0.3182
PRC1	rs8042680	15	C	NA	NA	NA
AP3S2	rs2028299	15	C	-0.2045	0.3786	0.5905
MAF	rs17797882	16	T	0.1923	0.325	0.5557
FTO	rs8050136	16	A	0.6776	0.3711	0.07169
SRR	rs391300	17	A	-0.1986	0.3296	0.5485
PEPD	rs3786897	19	G	0.3499	0.3244	0.2841
HNF4A	rs6017317	20	G	0.3117	0.3171	0.3286
HNF4A	rs4812829	20	A	0.1263	0.339	0.7105

Note:

Δ value=T*48 weeks* value - baseline (T0) value. Linear regression was under an additive model with adjustment for age, gender, BMI at baseline.

Supplementary Table 5 Association between SNPs and △2hPG after 48 weeks of treatment in the rosiglitazone cohort

Gene/location	SNP	Chromosome	Allele	BETA	SEM	*P* value
RBMS1	rs7593730	2	T	0.7744	0.9829	0.4333
near *GRB14*	rs3923113	2	G	-0.04312	0.961	0.9643
UBE2E2	rs7612463	3	A	-1.142	0.7832	0.1491
IGF2BP2	rs7651090	3	G	0.04727	0.681	0.9448
PPARG	rs1801282	3	G	-2.68	1.393	0.05822
ADAMTS9-AS2	rs4607103	3	T	-0.7879	0.6568	0.2341
ST6GAL1	rs16861329	3	T	0.1449	0.825	0.8611
MAEA	rs6815464	4	G	0.08084	0.6886	0.9069
WFS1	rs10010131	4	A	-1.304	1.467	0.3769
ZBED3-AS1	rs4457053	5	G	2.404	1.487	0.1101
ZFAND3	rs9470794	6	C	-0.5167	0.6757	0.4469
CDKAL1	rs7756992	6	A	-0.3359	0.6079	0.5822
KCNK16	rs1535500	6	T	-0.0376	0.592	0.9495
YKT6	rs917793	7	A	-0.6542	0.707	0.3578
KLF14	rs972283	7	A	1.087	0.6469	0.09719
JAZF1	rs864745	7	G	0.4088	0.5501	0.4598

PAX4	rs6467136	7	A	-2.036	0.8428	0.01819
TP53INP1	rs896854	8	A	0.2143	0.7053	0.7621
SLC30A8	rs13266634	8	T	-1.158	0.5999	0.05732
GLIS3	rs7041847	9	A	0.6457	0.6268	0.3063
PTPRD	rs17584499	9	T	2.175	1.231	0.08129
CHCHD9	rs13292136	9	T	-0.8552	1.146	0.458
10p13	rs10906115	10	G	0.06897	0.6621	0.9173
VPS26A	rs1802295	10	T	-0.6876	0.7837	0.3832
CDC123	rs12779790	10	G	-0.235	0.7623	0.7587
TCF7L2	rs7903146	10	T	1.497	1.643	0.3651
KCNQ1	rs231362	11	T	1.078	0.9365	0.2533
ARAP1	rs1552224	11	G	-0.2239	1.201	0.8526
KCNQ1	rs2237892	11	T	-0.2167	0.7283	0.7669
KCNJ11	rs5219	11	T	0.5562	0.6042	0.3603
TENM4	rs10751301	11	C	1.53	0.726	0.03845
TSPAN8	rs7961581	12	C	0.7832	0.6677	0.2446
OASL	rs7957197	12	T	NA	NA	NA
RPSAP52	rs1531343	12	C	1.397	1.026	0.1776

13q31.1	rs1359790	13	T	-0.1562	0.6687	0.8159
HMG20A	rs7178572	15	G	0.9134	0.557	0.1053
15q22.2	rs1436955	15	A	-0.8186	0.6933	0.2415
ZFAND6	rs11634397	15	G	-1.036	1.103	0.3506
PRC1	rs8042680	15	C	NA	NA	NA
AP3S2	rs2028299	15	C	0.5508	0.7613	0.4717
MAF	rs17797882	16	T	1.02	0.6425	0.1166
FTO	rs8050136	16	A	0.05392	0.7392	0.9421
SRR	rs391300	17	A	0.2036	0.6356	0.7496
PEPD	rs3786897	19	G	0.4435	0.644	0.4932
HNF4A	rs6017317	20	G	-0.4348	0.6134	0.4806
HNF4A	rs4812829	20	A	-0.7847	0.6396	0.2238

Note:

Δ value=T*48 weeks* value - baseline (T0) value. Linear regression was under an additive model with adjustment for age, gender, BMI at baseline.

Supplementary Table 6 Association between SNPs and △HbA1c levels after 48 weeks of treatment in the rosiglitazone cohort

**Gene/location**	**SNP**	**Chromosome**	**Allele**	**BETA**	**SEM**	***P* value**
RBMS1	rs7593730	2	T	0.2585	0.3974	0.5173
near *GRB14*	rs3923113	2	G	0.01684	0.378	0.9646
UBE2E2	rs7612463	3	A	-0.09247	0.3203	0.7736
IGF2BP2	rs7651090	3	G	0.05143	0.2765	0.8529
PPARG	rs1801282	3	G	-0.00278	0.5871	0.9962
ADAMTS9-AS2	rs4607103	3	T	0.03092	0.2719	0.9098
ST6GAL1	rs16861329	3	T	-0.00889	0.3316	0.9787
MAEA	rs6815464	4	G	-0.06573	0.2818	0.8162
WFS1	rs10010131	4	A	0.3674	0.6045	0.5451
ZBED3-AS1	rs4457053	5	G	0.345	0.6201	0.5796
ZFAND3	rs9470794	6	C	-0.2701	0.2861	0.3482
CDKAL1	rs7756992	6	A	0.2808	0.2394	0.2445
KCNK16	rs1535500	6	T	0.1794	0.2372	0.4517
YKT6	rs917793	7	A	-0.3329	0.2744	0.2289
KLF14	rs972283	7	A	-0.5836	0.2733	0.03591
JAZF1	rs864745	7	G	-0.1887	0.2229	0.4

PAX4	rs6467136	7	A	-0.203	0.3603	0.5748
TP53INP1	rs896854	8	A	0.52	0.2714	0.05914
SLC30A8	rs13266634	8	T	-0.07683	0.2506	0.76
GLIS3	rs7041847	9	A	0.05729	0.2577	0.8246
PTPRD	rs17584499	9	T	0.7574	0.5212	0.1503
CHCHD9	rs13292136	9	T	0.07744	0.4518	0.8643
10p13	rs10906115	10	G	0.03675	0.2608	0.8883
VPS26A	rs1802295	10	T	0.3045	0.3289	0.3575
CDC123	rs12779790	10	G	0.134	0.2994	0.6558
TCF7L2	rs7903146	10	T	-0.1222	0.629	0.8465
KCNQ1	rs231362	11	T	0.02935	0.3804	0.9387
ARAP1	rs1552224	11	G	-0.5239	0.4854	0.2838
KCNQ1	rs2237892	11	T	-0.2005	0.2941	0.4974
KCNJ11	rs5219	11	T	0.1596	0.2485	0.5227
TENM4	rs10751301	11	C	-0.2147	0.2965	0.4713
TSPAN8	rs7961581	12	C	-0.05885	0.2822	0.8354
OASL	rs7957197	12	T	NA	NA	NA
RPSAP52	rs1531343	12	C	-0.759	0.4262	0.07898

13q31.1	rs1359790	13	T	-0.04908	0.2714	0.857
HMG20A	rs7178572	15	G	0.5013	0.2234	0.02775
15q22.2	rs1436955	15	A	-0.09526	0.2855	0.7395
ZFAND6	rs11634397	15	G	-0.3452	0.4479	0.4433
PRC1	rs8042680	15	C	NA	NA	NA
AP3S2	rs2028299	15	C	0.0109	0.302	0.9713
MAF	rs17797882	16	T	0.4025	0.2552	0.1188
FTO	rs8050136	16	A	0.2282	0.3032	0.454
SRR	rs391300	17	A	0.1021	0.2621	0.6979
PEPD	rs3786897	19	G	0.08857	0.2685	0.7424
HNF4A	rs6017317	20	G	0.203	0.2556	0.4297
HNF4A	rs4812829	20	A	0.08527	0.2581	0.7421

Note:

Δ value=T*48 weeks* value - baseline (T0) value. Linear regression was under an additive model with adjustment for age, gender, BMI at baseline.
